# Effects of immersive virtual reality training on the adaptive skills of children and adolescents with high functioning autism spectrum disorder: a mixed-methods pre-post study

**DOI:** 10.3389/fpsyt.2025.1570437

**Published:** 2025-08-18

**Authors:** Wenjie Yan, Tianyu Zhai, Yan Li, Jing Chen, Zhixin Sun, Yuxiong Lin, Xiyan Zhang, Yasong Du

**Affiliations:** ^1^ Shanghai Mental Health Center, Shanghai Jiao Tong University School of Medicine, Shanghai, China; ^2^ Clinical Research Center for Mental Disorders, Shanghai Pudong New Area Mental Health Center, School of Medicine, Tongji University, Shanghai, China

**Keywords:** immersive virtual reality, autism spectrum disorder, adaptive skill, daily life skill, usability

## Abstract

**Background:**

Autism Spectrum Disorder (ASD) is characterized by social deficits and restricted, repetitive behaviors, with fewer than 10% achieving independent adulthood. Immersive virtual reality (IVR) provides a novel training approach through interactive and realistic environments. This study developed an IVR system to enhance adaptive skills in children and adolescents with high-functioning ASD.

**Methods:**

Thirty-three individuals with high-functioning ASD (ages 8–18) were enrolled based on clinical diagnoses confirmed by Diagnostic and Statistical Manual of Mental Disorders, Fifth Edition (DSM-5) and Autism Diagnostic Interview-Revised (ADI-R), with an intelligence quotient ≥ 80. The study employed a single-arm, within-subject pre-post design. Participants received weekly one-hour IVR training sessions, requiring 6–10 sessions to complete 36 tasks twice. Primary outcomes included changes in IVR task scores and completion times, while secondary outcomes assessed parent-reported questionnaires — Adaptive Behavior Assessment System-Second Edition (ABAS-II), Autism Behavior Checklist (ABC), and Behavior Rating Inventory of Executive Function (BRIEF) — neuropsychological tests (Go/No-Go, 0-back, 1-back, and emotional face recognition tasks), and semi-structured interviews. Usability was evaluated via self-reported comfort levels, willingness to continue and device-related questions. Generalized Estimating Equation models analyzed changes across all measures.

**Results:**

The IVR training demonstrated high usability, with an 87.9% completion rate and no severe adverse effects. Some participants reported mild discomforts, including dizziness (28.6%) and fatigue (25.0%), as well as device-related issues such as unsteady walking (34.5%) and headset discomfort (31.0%). However, comfort levels increased over time (adjusted *P* = 0.026), indicating gradual adaptation to the system. IVR task scores improved by 5.5% (adjusted *P* = 0.034), and completion times decreased by 29.59% (adjusted *P* < 0.001). Parent-reported measures showed a 43.22% reduction in ABC Relating subscale scores (adjusted *P* = 0.006) and moderate reductions in BRIEF Behavioral Regulation and Metacognition indices (adjusted *P* = 0.020, 0.019). Reaction times for the 1-back and emotional face recognition tasks decreased by 14.81% and 14.14% respectively (adjusted *P* = 0.012, 0.014). Qualitative feedback indicated improvements in social interaction, emotional regulation, repetitive behaviors, attention, and daily living skills.

**Conclusion:**

This pilot study supports IVR training as a feasible and potentially effective training method for improving adaptive skills in children and adolescents with high functioning ASD.

## 1 Introduction

Autism Spectrum Disorder (ASD) is a neurodevelopmental condition manifested predominantly by persistent deficits in social communication and social interaction as well as limited, restricted, repetitive patterns of behavior, interests, or activities (1). Epidemiological studies indicate that between 1990 and 2019, the global prevalence of ASD increased steadily in countries with a high socio-demographic index (2). Among 8-year-old children in the United States, ASD prevalence rates were reported as 1.46% in 2012 (3), 1.68% in 2014 (4), 2.30% in 2018 (5), and 2.76% in 2020 (6). In China, the prevalence of ASD among children aged 6–12 is approximately 0.7% (7). Notably, the prevalence among boys (1.0%) is significantly higher than that among girls (0.2%) (8).

Deficits in adaptive functioning are a key feature of ASD and represent a significant contributor to functional impairments in this population. Adaptive functioning refers to the ability to perform daily activities required to meet personal and societal demands, covering three domains: communication, socialization, and daily living skills (9). Unlike cognitive or intellectual abilities, which represent an individual’s theoretical capacity to perform specific tasks, adaptive functioning reflects the practical ability to execute those tasks independently and without external assistance. Although 49%–69% of individuals with ASD have normal intellectual functioning (intelligence quotient, IQ ≥ 80) (6), labeling these individuals as “high-functioning” might be misleading. This term overlooks the substantial challenges they may encounter in areas such as independent living, employment, and social relationships (10). Such challenges highlight the critical need to address deficits in adaptive functioning, even in individuals with higher cognitive potential.

Research underscores the pivotal role of adaptive skills—particularly daily living skills—in determining long-term outcomes for individuals with ASD (11). For instance, delays in adaptive skills at ages 4 and 9 have been shown to correlate with poorer adult outcomes (12), while higher levels of adaptive functioning are associated with more positive trajectories (13). Improved adaptive skills not only enhance self-care but also reduce dependency, improve quality of life, and lower social costs (14–16). Hence, trainings targeting the adaptive skills of children and adolescents with ASD, even in individuals with theoretically stronger cognitive abilities, are exceptionally valuable to support a successful transition to independent living adulthood.

Virtual Reality (VR) technology uses algorithms to create three-dimensional environments that simulate and enhance sensory experiences by mirroring changes in the physical world. Based on the level of immersion and presentation modality, VR is typically categorized as non-immersive (desktop-based), semi-immersive (cave-based), and fully immersive (headset-based). However, this classification mainly reflects technical implementation and does not fully capture the actual immersive experience perceived by users. Immersion is a multidimensional construct influenced by display type, system performance, interaction quality, user engagement, the virtual environment’s visual style, among other factors. In practical applications, non-immersive VR has been widely adopted in training adaptive skills among children and adolescents with ASD, largely due to its lower cost and ease of implementation. For instance, a well-developed desktop VR has been used to enhance job interview skills in adolescents with ASD, improving performance and reducing anxiety (17–19). Similarly, Lamash et al. employed desktop-based VR for grocery shopping training, demonstrating significant improvements in attention, executive function, shopping accuracy, and strategy use, with these skills transferring effectively to real-world settings (20). Furthermore, it has also been applied to train bus-taking skills (21), among other adaptive behaviors, highlighting its versatility.

Unlike desktop-based and cave-based VR, which provide three-dimensional visualization on two-dimensional screens or room walls (22), immersive VR (IVR) is typically associated with head-mounted displays (HMDs). These HMDs integrate visors equipped with small cathode ray tubes or liquid-crystal displays, combined with optical augmentation, producing a virtual screen perceived as spatially distant by users (23). IVR offers distinct advantages due to its interactivity, immersion, and ability to evoke a heightened sense of “presence”—the subjective perception of being physically present in a virtual environment (24). This sense of presence enables individuals to exhibit emotions and behaviors that similar to those in real-world contexts (25). Compared to non-immersive VR, IVR offers greater ecological validity, making it particularly beneficial for individuals with ASD, who often struggle with generalization—the ability to transfer learned skills to new settings (26). Traditional training methods often fail when real-life situations diverge from structured training scenarios, limiting the application of acquired skills (27). IVR addresses these limitations by creating realistic and immersive scenarios that mimic real-world conditions, facilitating skill generalization. For example, Dixon et al. developed an IVR system for training street-crossing safety, where children with ASD achieved mastery standards in both virtual and real-world settings following the training (28). Similarly, Miller et al. designed an IVR system for air travel training, which improved ASD children’s vocabulary and language skills related to flight boarding (29, 30).

An innovative feature of this study is the integration of a treadmill with the IVR system, in addition to the traditional headset and handheld controllers. The treadmill, equipped with multidirectional wheels, allows participants to physically walk within the virtual environment towards any directions, replacing button-based navigation. This addition enhances realism and immersion, especially in scenarios requiring extensive movement. Building on this hardware innovation, we designed a comprehensive IVR training system to target multiple adaptive skills in children and adolescents with high functioning ASD. Four key scenarios were selected to address essential daily skills: subway, supermarket, home, and amusement park.

The subway scenario replicates Shanghai’s subway system, including routes, stations, ticket machines, security checkpoints, and train interiors, along with auditory elements such as announcements and door-closing sounds, etc. This design allows participants to practice safe and independent subway travel through repeated exercises. The supermarket scenario focuses on critical shopping skills, such as using shopping carts, weighing items, and managing flexible payment methods like QR codes, Alipay, and cash. The environment is modeled after local supermarkets to improve familiarity and skill transfer. In contrast, the home and amusement park scenarios offer more varied tasks. The home scenario trains independent living skills, including organizing backpacks, sorting trash, cleaning utensils, selecting clothing, and identifying potential hazards, etc. The amusement park scenario maintains participant engagement while simulating social interactions, such as greeting friends, discussing activities, dining in restaurants, resolving conflicts, and navigating challenges like locating a lost companion. Each task requires participants to remember and follow instructions, which is expected to enhance executive function, particularly working memory. Tasks also involve spatial judgment and navigation, with the amusement park scenario particularly incorporating map-based guidance. These design features aim to improve both executive function and spatial processing.

In summary, the primary objective of this study was to enhance multiple adaptive skills in children and adolescents with high functioning ASD through IVR training. Task scores and completion times recorded by the IVR system served as the primary outcome measures, given the lack of specialized tools to directly evaluate the targeted skills. Secondary outcomes included parent-reported questionnaires (for evaluating adaptive skills, autism symptoms, and executive skills), caregivers’ interviews (for validation of questionnaires), and neuropsychological tests (for objectively measuring changes in executive function and emotion recognition). Additionally, participants’ comfort levels and willingness to continue training after each session, as well as their responses to questions regarding device usage at the end of the final session, were recorded to assess system usability.

## 2 Materials and methods

### 2.1 Participants

Participants were recruited between September 2023 and August 2024. Inclusion criteria were (1): aged 8–18 years (2); diagnosed with ASD according to DSM-5 criteria by at least one psychiatrist or pediatrician at the level of associate chief or above, in conjunction with meeting the diagnostic thresholds of the Autism Diagnostic Interview-Revised (ADI-R) (31) (3); with full-scale intelligence quotient (FSIQ) of the Wechsler Intelligence Scale of 80 or above (32, 33) (4); willingness to participate in the study, with the ability to understand instructions and complete follow-up assessments within the specified timeframe (5); if undergoing other treatments at the time of enrollment, maintaining an unchanged treatment regimen for four weeks before and throughout the study. Exclusion criteria included visual or hearing impairments, epilepsy, severe physical illnesses, organic brain diseases, schizophrenia, depressive disorders, anxiety disorders, or any other neurological and psychiatric conditions deemed unsuitable for IVR training.

The study was approved by the Ethics Committee of Shanghai Mental Health Center (Approval No. 2019-02) to guarantee that all necessary ethical requirements were met, and the informed consent was signed voluntarily by each participant and guardian in prior. Participants’ personal data, including any information and the metrics collected during the training, were kept strictly confidential. All costs associated with the IVR training and assessments were fully covered by the researcher.

### 2.2 IVR training

#### 2.2.1 Hardware setup

**Figure 1:**
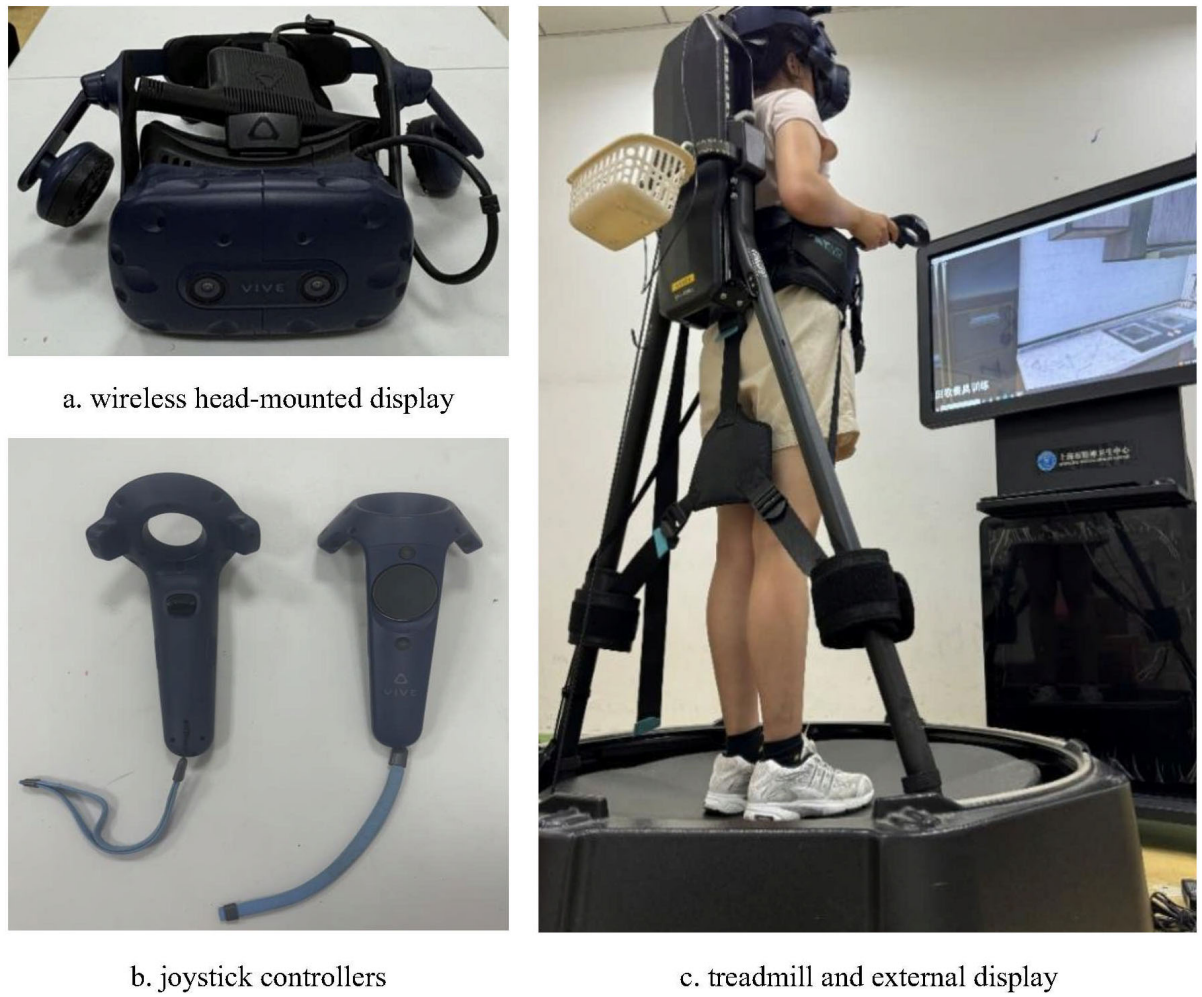
Hardware setup.

A wireless head-mounted display (HMD) was used to display immersive virtual scenes to the participant in real time. Two joystick controllers enabled interaction with the virtual environment, allowing actions such as option selection and grasping. A multidirectional treadmill equipped with omni-wheels allowed participants to walk or run freely in any direction, depending on task requirements. For safety, participants were secured with a harness and positioned within a central safety frame on the treadmill. When participants’ head rotated or moved, the head of their avatar in the virtual environment simultaneously rotated by the same angle or moved the same distance in the corresponding direction, with head movement tracked via the HMD. Pressure sensors embedded in the base of the treadmill detected stepping motions; thus, when participants marched in place, forward walking in the virtual space was simulated at a frequency matched to their stepping rate. The initial step length was preset to 0.8 meters per step. During the preparation phase in the first session, participants were asked to confirm the appropriateness of this step length, which was adjusted if necessary. Additionally, an external display was used by researchers to observe participants’ real-time first-person view and to provide task-related guidance when needed (see **Figure 1**, **Supplementary Video 1**).

#### 2.2.2 Training scenarios

**Figure 2:**
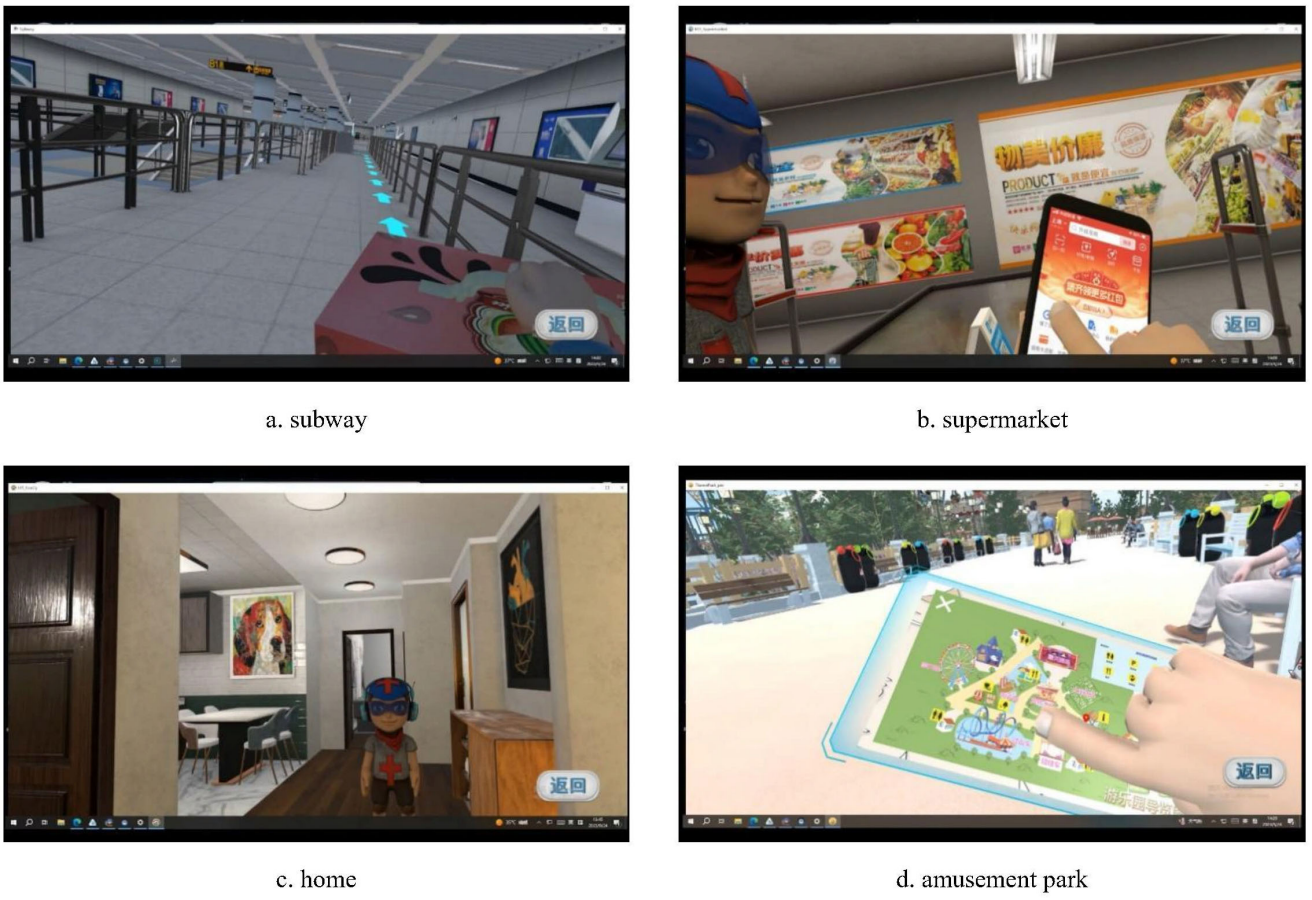
Demonstration of four scenarios.

The virtual training environment was developed based on the SteamVR platform and comprised a total of 36 tasks across four scenarios: subway (10 tasks), supermarket (9 tasks), home (9 tasks), and amusement park (8 tasks) (see **Figure 2**, **Table 1**).

**Table 1:** Overview of training scenarios.

Scenarios	Training content	Task requirements	Virtual characters and dialogue examples
Subway	Covers essential subway taking skills and instrumental interaction skills, including ticket purchase, security checks, boarding, route planning, transfers, handling unexpected situations such as missing a station, etc.	Each task starts with voice instructions from a virtual mother, and participants are required to complete the task accordingly. To ensure that participants understand the task procedure at a theoretical level beforehand, the system first presents a set of questions on a virtual panel—such as which subway line to take and where to transfer. Following this, participants proceed to perform the task by simulating the real-life process of riding the subway.	Mother: Provides task instructions, e.g., “You’re now at Dong’an Road Station, you need to take Line 4 and get off at Shanghai Indoor Stadium Station. I’ll be waiting for you at Exit 2.” Security Officer: Provides guidance on correct security procedures, e.g., “Excuse me, balloons are not allowed in the station.”
Supermarket	Focuses on essential shopping skills and instrumental interaction skills, such as selecting individual, bulk, and mixed items, using shopping carts or self-checkout, seeking help when necessary, etc.	Each task starts with voice instructions from a virtual mother, and participants are required to complete the task accordingly. If participants forget the items to be purchased or their quantities, they can refer to a virtual panel located on the avatar’s left hand to recall the instructions.	Mother: Provides task instructions, e.g., “I’d like you to help buy 3 bottles of mineral water, 5 apples, and 3 pears.” Cashier: Assists with the checkout process, e.g., “That’s 10 yuan in total. How would you like to pay?” Weighing Staff: Assists with weighing items. Store Assistant: Provides help when participants request assistance.
Home	Aims at developing daily living skills and affective interactions skills, including attention, object identification, organizing a backpack, sorting trash, identifying hazards at home, etc.	Each task begins with either voice instructions from a virtual mother or a request from a virtual friend “Xiao Zhi”, and participants are required to complete the task accordingly. For certain tasks, to ensure that participants understand the procedure at a theoretical level, the system presents a set of questions on a virtual panel located on the avatar’s left hand—for example, how to handle hazardous items.	Mother: Provides task instructions, e.g., “It’s cloudy today and drizzling outside. What kind of clothes should you wear when going out?” Xiao Zhi: Provides task instructions, e.g., “Show me around your house!”
Amusement park	Focuses on navigation skills and affective and instrumental interaction skills, such as purchasing tickets, locating attractions, buying souvenirs, addressing emergencies like losing a companion or resolving conflicts, etc.	Each task starts with an interaction or request initiated by the virtual friend “Xiao Zhi.” Participants are required to engage with Xiao Zhi through options presented on a virtual panel and then use the map located on the avatar’s left hand to identify the target location and complete the task. For example, in the ticket-purchasing task, participants must first greet Xiao Zhi by selecting panel options, then locate the ticket hall using the map, and finally complete the ticket purchase by selecting panel options to interact with the ticket clerk.	Xiao Zhi: Provides task instructions and interacts with participants, e.g., “It’s already 12 p.m., let’s go have lunch.” Staff: Engages in location-specific interactions with participants, such as ticket clerks, gate inspectors, or restaurant receptionists. Bystanders: Engage in situational interactions with participants, such as standing and obstructing the participant’s view during a theater performance.

**Figure 3:**
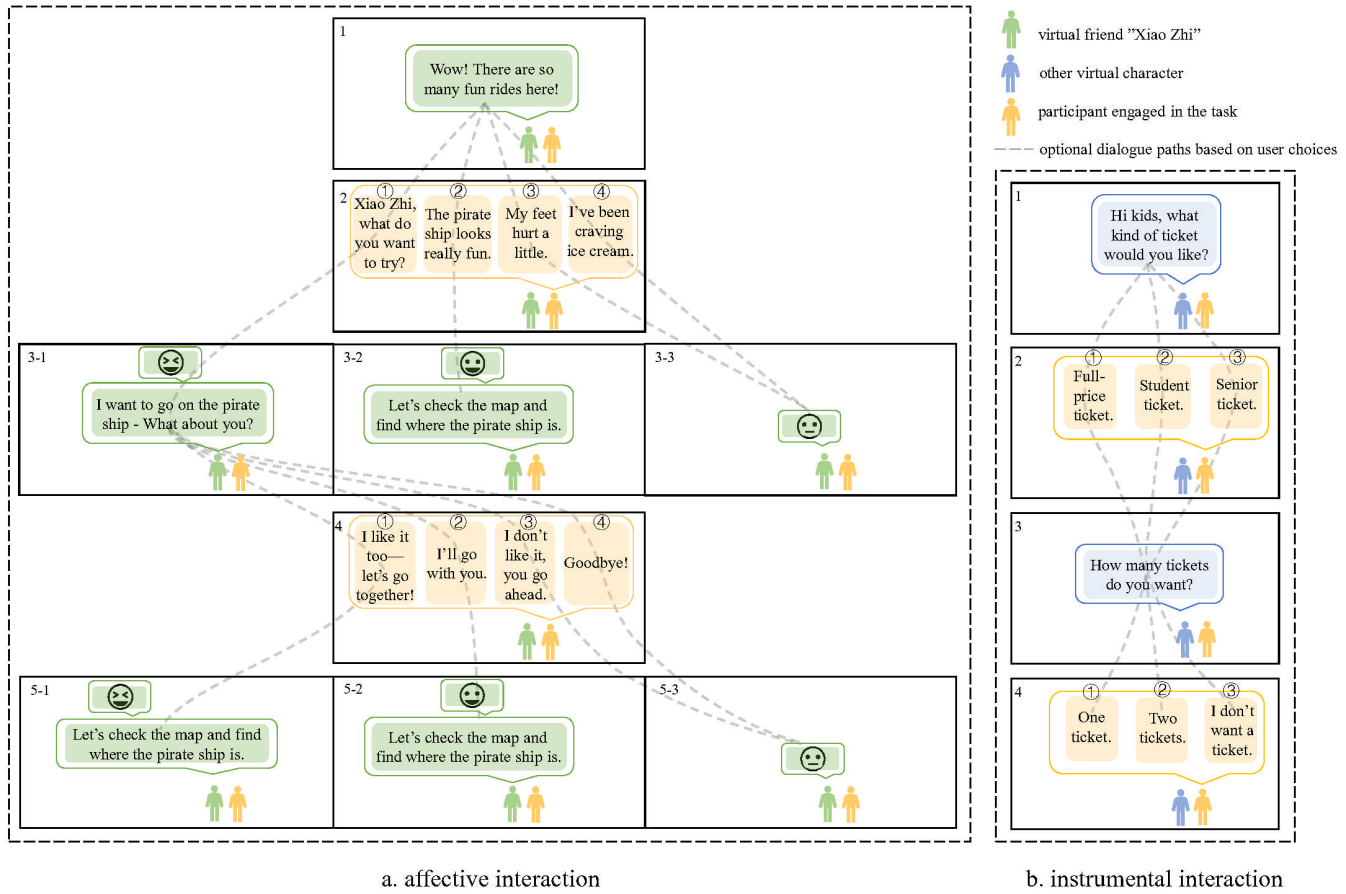
Schematic of participant–virtual character interaction.

Each task was guided by virtual characters operating on preprogrammed scripts (see **Figure 3**). Among them, the virtual character “Xiao Zhi,” a boy with short black hair wearing a blue shirt, primarily appeared in the home and amusement park scenarios to facilitate affective interactions. Other virtual characters were designed to support instrumental interactions based on the specific task context—for example, a security officer at the subway security checkpoint or a cashier at the supermarket checkout counter.

#### 2.2.3 Training procedure

**Figure 4:**
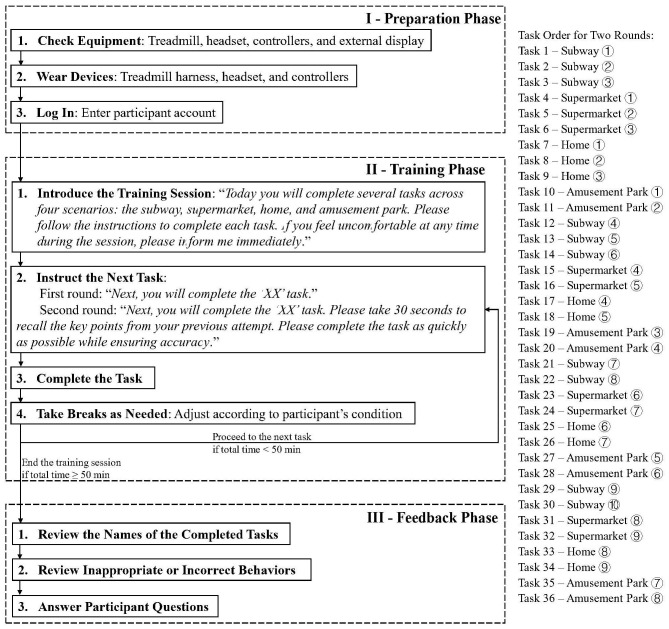
Training procedure flowchart.

During the entire training period, each participant was required to complete all 36 tasks twice, following a predetermined sequence from Task 1 to Task 36. After completing the first full round of tasks, participants repeated the same tasks to reinforce the skills acquired during the initial round of training. Each training session was designed to last approximately 60 minutes and was conducted once per week. The structure of each session included: 5 minutes of preparation phase, 50 minutes of training phase (including short breaks between tasks), and 5 minutes of feedback phase (see **Figure 4**). Each training session resumed from where the previous session ended. Given the variability in baseline abilities and responsiveness to training among participants, the time required to complete each task varied across individuals. Consequently, the total number of sessions needed to complete the entire training differed among participants. On average, participants required eight sessions to complete the program, with the number of sessions ranging from six to ten. Notably, all participants completed the same total number of tasks.

### 2.3 Measurements

#### 2.3.1 Training completion and acceptability

At the end of each training session, participants verbally rated their willingness to continue training on a scale from 1 (not at all willing) to 7 (very willing) and their comfort level on a scale from 1 (very uncomfortable) to 7 (very comfortable). Ratings were recorded at three time points: after the first session (T1), after the first round of training (T2, approximately midway through all sessions), and after the second round (T3, the final session). At T3, participants were additionally asked whether they had experienced any of the following issues during the training sessions (1): discomfort while wearing the device (2), unstable connection (3), poor experience quality (4), dizziness or nausea (5), headache or fatigue (6), unsteady walking or falls (7), irritability, anxiety, or fear, or (8) other concerns.

#### 2.3.2 IVR performance

Task scores and completion times are automatically tracked by the IVR system. Scores are determined by in-task behaviors, with a total maximum score of 127 points across all scenarios. The subway scenario has a maximum of 21 points, scored on destination selection, route choice, and risky behavior avoidance. The supermarket scenario has a maximum of 32 points, scored on task memory and target item selection. The home scenario has a maximum of 37 points, scored on task accuracy and attempts. Similarly, the amusement park scenario has a maximum of 37 points, scored on task completion and attempts. Completion time is measured from task initiation to completion, including system instructions but excluding breaks, equipment handling, and loading times.

#### 2.3.3 Quantitative parent-reported survey

The Adaptive Behavior Assessment System-II (ABAS-II) evaluates an individual’s capacity to adapt to daily life demands through three main domains: conceptual skills (communication, functional academics, self-direction), social skills (leisure, social), and practical skills (community use, home living, health and safety, self-care) (34). A General Adaptive Composite (GAC) score ≥ 80 is considered normal.

The Autism Behavior Checklist (ABC) is a widely used tool for assessing autistic behaviors and is often employed to monitor behavioral changes over time (35). The checklist includes 57 items across five subscales: sensory, relating, body and object use, language, and social and self-help skills. Higher scores suggest a greater likelihood of autism.

The Behavior Rating Inventory of Executive Function (BRIEF) consists of 63 items that assess two dimensions of executive functioning (36): the Behavioral Regulation Index (BRI), which includes inhibition, shifting, and emotional control, and the Metacognition Index (MI), which evaluates initiation, working memory, planning/organization, organization of materials, and task monitoring. A T-score of 50 is considered average, with scores ≥ 65 indicating clinically significant difficulties in executive functioning.

The outcome variables were derived from the subscale scores of the questionnaires.

#### 2.3.4 Neuropsychological test

The neuropsychological tests were designed and presented using E-Prime 3.0 software. The paradigms included the go/no-go task, 0-back task, 1-back task, and emotional face recognition task. Detailed task designs can be found in **Supplementary Material 1**. Reaction times and accuracy during the testing were automatically recorded by E-Prime. Participants were required to complete practice trials before the formal testing, with a minimum accuracy of 60% required to proceed. All testing took place in a same quiet room.

#### 2.3.5 Qualitative interview

The self-constructed interview included the question: “Have you noticed improvements in any skills after the IVR training?”

### 2.4 Procedure

**Figure 5:**
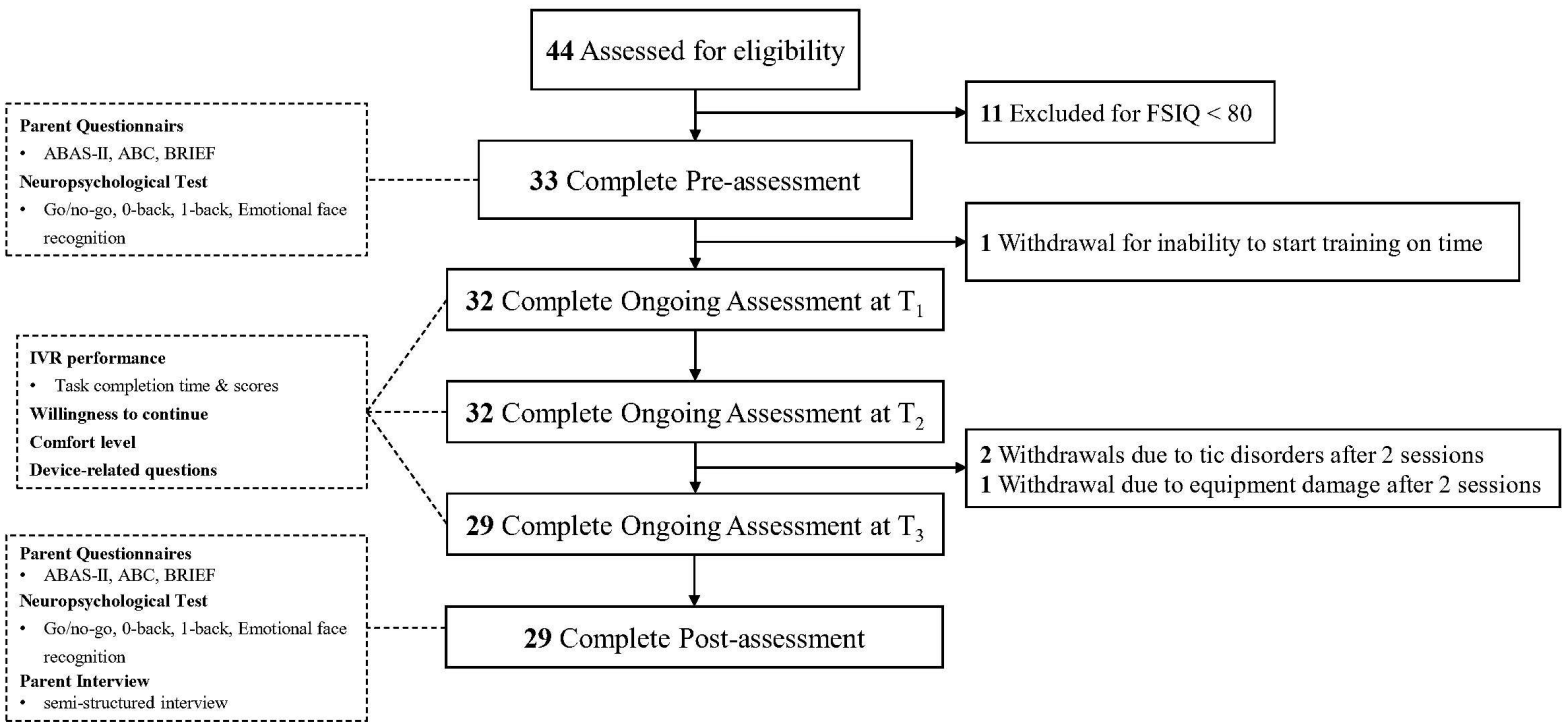
Flowchart of the study.

See **Figure 5** for the flowchart of the study. Participants were recruited from the Shanghai Mental Health Center (SMHC) and via online posters. After confirming the clinical diagnosis, eligible individuals were invited to SMHC to provide informed consent and undergo the Wechsler Intelligence Scale test. Participants with an FSIQ ≥ 80 completed pre-assessments, including parent questionnaires and neuropsychological tests. The IVR training commenced within one week of completing the assessments. During each session, IVR performance, willingness to continue, and comfort level were recorded.

Post-assessments, including parent questionnaires, neuropsychological tests, and a semi-structured interview, were conducted within one week after training. All assessments and IVR training were completed within three months, with the same researcher conducting all training sessions to ensure consistency. To maintain objectivity, interviews and assessments were carried out by an independent researcher who was not involved in the training process.

### 2.5 Data analysis

Statistical analyses were performed using SPSS (version 25.0) with an intent-to-treat (ITT) approach, including all participants who complete baseline assessment. Baseline demographic and clinical characteristics were summarized using means and standard deviations for continuous variables that followed a normal distribution, while categorical variables were described as counts and frequencies.

Generalized Estimating Equations (GEE) models were employed to assess changes in comfort level and willingness to continue during training sessions, as well as changes in task completion times and scores between the first and second rounds, as well as parent-reported questionnaire scores, and reaction times from neuropsychological tests before and after training. The GEE models adjusted for age, gender, FSIQ, and additional treatment status, with further correction for task accuracy and key press reaction time in neuropsychological test reaction times. Estimated marginal means and their standard errors were derived from the models, and these were further used to calculate the percentage change and Cohen’s d effect sizes. A p-value of less than 0.05 was considered statistically significant. Given the multiple comparisons made, Bonferroni correction was applied to adjust the results for each dimension (Willingness to Continue, Comfort Level, IVR Performance, Parent Questionnaires, and Neuropsychological Test).

For qualitative analysis, the interviews were transcribed verbatim using Tencent Meeting and analyzed using the framework approach. Two researchers independently coded the transcripts, refined the themes, and translated the themes and quotations from Chinese to English.

## 3 Results

### 3.1 Participants characteristics

See **Figure 5** for the flowchart of the study. During the recruitment period, 44 individuals registered to participate in the study. Of these, 11 were excluded due to an FSIQ below 70, and 33 completed the baseline assessments and proceeded with the training.

See **Table 2** for baseline demographic characteristics of 33 participants. Most participants were male (n = 28, 84.6%) and adolescents (n = 19, 57.6%), with 63.6% (n = 21) receiving additional treatments. Regarding family characteristics, the majority were cared for by their mothers (n = 27, 81.8%), and the primary caregiver had a tertiary education (n = 25, 75.8%). Over half of the families had an annual income exceeding 300,000 CNY (n = 19, 57.6%), and most participants were only children (n = 20, 60.6%). Extended family living arrangements were common (n = 22, 66.7%), and family relationships were mostly rated as moderate (n = 17, 51.5%).

**Table 2:** Baseline demographic characteristics (N=33).

Variables	N (%)
Gender	
Male	28 (84.6)
Female	5 (15.2)
Age	13.7 (2.8)
Children (8–12 years)	14 (42.4)
Adolescents (13–18 years)	19 (57.6)
FSIQ	103.5 (15.4)
Additional treatment	
Yes	21 (63.6)
No	12 (36.4)
Primary caregiver	
Mother	27 (81.8)
Others	6 (18.2)
Educational attainment of the primary caregiver	
Secondary education	8 (24.2)
Tertiary education	25 (75.8)
Annual household income	
≤300,000 CNY/year	14 (42.4)
>300,000 CNY/year	19 (57.6)
Only child status	
Yes	20 (60.6)
No	13 (39.4)
Family composition	
Nuclear family	2 (6.1)
Extended family	22 (66.7)
Other family types	9 (27.3)
Quality of family relationships	
Harmonious	11 (33.3)
Moderate	17 (51.5)
Strained	5 (15.2)

### 3.2 Training completion and acceptability

See **Figure 5** for the completion and dropout of the training. Among the 33 participants, 1 (3.0%) withdrew due to an inability to start the training on time, 2 (6.1%) withdrew after 2 sessions because tic symptoms significantly interfered with headset use, and 1 (3.0%) withdrew after 2 sessions due to repeated damage to the equipment. Of the remaining participants, 11 (33.3%) completed all tasks in 6 sessions, 6 (18.2%) in 7 sessions, 9 (27.3%) in 8 sessions, 1 (3.0%) in 9 sessions, and 2 (6.1%) in 10 sessions. For the attrition analysis, the characteristics of the 33 participants who completed baseline assessments were compared with those of the 29 participants who completed all training and assessments. No significant differences were found, indicating that attrition occurred randomly.

See **Table 3** for a comparative analysis of acceptability across different time points during training. At T1, the comfort level was 4.89. There was a general upward trend in comfort level across the three time points during the training, with a significant time effect from T2 to T3 (adjusted *P* = 0.026), and the effect size of 0.48 indicated a moderate effect. The willingness to continue at T1 was 6.13, and although fluctuations were observed throughout the training, no significant time effect was found.

**Table 3:** Changes in the comfort level and willingness to continue during training based on the generalized estimating equation model (N=33).

Outcome Variables	T_1_	T_2_	T_3_	Comparison	β (95%CI)	Adjusted *P* value	Percent change	Cohen’s d
Comfort Level	4.89 (0.21)	4.92 (0.20)	5.27 (0.16)	T_2_ to T_1_	0.04 (0.16~0.35)	1.000	0.79	0.06
				T_3_ to T_1_	0.38 (0.06~0.70)	0.057	7.85	0.43
				T_3_ to T_2_	0.34 (0.09~0.60)	0.026	7.00	0.48
Willingness to Continue	6.13 (0.17)	5.81 (0.26)	6.05 (0.18)	T_2_ to T_1_	-0.31 (-0.77~0.14)	0.523	5.14	0.25
				T_3_ to T_1_	-0.07 (-0.37~0.22)	1.000	1.20	0.09
				T_3_ to T_2_	0.24 (-0.04~0.52)	0.277	4.15	0.31

The Generalized estimating equation model adjusted for gender, age, FSIQ, and additional treatment status. Variables are presented as estimated marginal means and standard errors, with the P-adjusted values corrected using the bonferroni method.

T1 refers to the time point at the end of the first training session; T2 refers to the time point at the end of the first round of training sessions; T3 refers to the time point at the end of the second round of training sessions, which also marks the final training session.

After the final session, 29 participants responded to questions regarding device usability: discomfort while wearing the device was reported by 9 (31.0%) participants, unstable connections by 8 (27.6%), poor experience quality by 3 (10.3%), dizziness or nausea by 8 (27.6%), headaches or fatigue by 7 (24.1%), unsteady walking or falls by 10 (34.5%), and other concerns, specifically frequent software bugs by 2 (6.9%).

### 3.3 IVR performance

See **Table 4** for changes in IVR task completion times and scores. From the first to the second round of training, task scores increased significantly by 5.5% (adjusted *P* = 0.034), with a moderate effect size (Cohen’s d = 0.55). Task completion time decreased significantly by 29.59% (adjusted *P* < 0.001), showing a large effect size (Cohen’s d = 2.19).

**Table 4:** Changes in the outcomes variables across time based on the generalized estimating equation model (N=33).

Outcome Variables	Pre/First round	Post/Second round	β (95%CI)	Adjusted *P* value	Percent change	Cohen’s d
IVR Performance						
Task Score	86.43 (2.01)	91.18 (0.36)	4.75 (1.57~7.93)	0.034	5.50	0.55
Completion Time	6488.39 (201.81)	4568.29 (111.66)	-1920.11 (-2244.61~-1595.60)	<0.001	29.59	2.19
Parent Questionnaires						
ABAS-II						
conceptual	86.19 (2.33)	89.07 (2.61)	2.86 (0.16~5.59)	0.381	3.33	0.42
social	76.38 (2.24)	76.14 (2.65)	-0.25 (-4.48~3.98)	1.000	0.33	0.02
practical	86.00 (2.31)	87.13 (2.44)	1.13 (-2.70~4.96)	1.000	1.31	0.12
ABC						
sensory	4.58 (0.94)	2.61 (0.69)	-1.97 (-3.94~0.00)	0.501	43.01	0.40
relating	10.55 (1.55)	5.99 (1.00)	-4.56 (-7.18~-1.95)	0.006	43.22	0.70
body and object use	6.08 (0.87)	3.34 (0.76)	-2.73 (-4.86~-0.61)	0.117	44.90	0.51
language	4.27 (0.87)	3.09 (0.72)	-1.18 (-3.15~ 0.79)	1.000	27.63	0.24
social and self-help skills	8.73 (1.09)	7.30 (1.00)	-1.42 (-3.62~ 0.77)	1.000	16.27	0.26
BRIEF						
BRI	66.85 (1.97)	61.52 (2.45)	-5.33 (-8.71~ -1.95)	0.020	7.97	0.63
MI	62.09 (1.79)	57.39 (2.10)	-4.69 (-7.66~-1.73)	0.019	7.55	0.63
Neuropsychological Test RT						
Go/no-go	422.04 (7.91)	410.81 (6.63)	-11.23 (-27.53~5.08)	0.885	2.66	0.25
0-back	505.87 (20.58)	466.08 (21.65)	-39.79 (-90.75~11.16)	0.63	7.87	0.29
1-back	580.69 (30.28)	494.66 (25.05)	-86.03 (-141.79~-30.27)	0.012	14.81	0.56
Emotional face recognition	1544.83 (70.88)	1326.43 (64.63)	-218.40 (-361.82~-74.97)	0.014	14.14	0.57
Mental rotation	1268.77 (98.77)	1003.67 (50.55)	-265.10 (89.10)	0.015	20.89	0.56

In the generalized estimating equation analysis, IVR performance and parent questionnaires were adjusted for age, gender, FSIQ, and additional treatment status. Neuropsychological test RT was adjusted for age, gender, FSIQ, additional treatment status, task accuracy and key press reaction time. Variables are presented as estimated marginal means and standard errors, with the P-adjusted values for IVR performance, parent questionnaires, and neuropsychological test RT corrected using the bonferroni method.

Pre refers to the baseline assessment, conducted one week prior to the training. First Round refers to the completion of all 36 IVR tasks during the first round. Second Round refers to the completion of all 36 IVR tasks during the second round. Post refers to the assessment conducted one week after the training ended. The unit for IVR performance total time is seconds (s); the unit for neuropsychological test RT is milliseconds (ms).

IVR, Immersive Virtual Reality; ABAS-II, Adaptive Behavior Assessment System-II; ABC, Achenbach Behavior Checklist; BRIEF, Behavior Rating Inventory of Executive Function; BRI, behavioral regulation index; MI, metacognition index; RT, Reaction Time.

### 3.4 Parent questionnaires

See **Table 4** for changes in parent-reported questionnaires. Notable improvements in social and executive functioning were observed in parent assessments. In the ABC scale, the Relating subscale score declined from a pre-training mean of 10.55 to 5.99 post-training (43.22% reduction, adjusted *P* = 0.006), with a moderate effect size (Cohen’s d = 0.70). The Behavioral Regulation Index of the BRIEF decreased from 66.85 to 61.52 (7.97% reduction, adjusted *P* = 0.020), with a moderate effect size (Cohen’s d = 0.63). Similarly, the Metacognition Index dropped from 62.09 to 57.39 (7.55% reduction, adjusted *P* = 0.019), also showing a moderate effect size (Cohen’s d = 0.63). No significant improvements were observed in the three subscales of the ABAS-II or other four ABC subscales, except for Relating.

### 3.5 Neuropsychological test

See **Table 4** for changes in reaction times across five neuropsychological tests. Reaction times for the 1-back task (14.81% reduction, adjusted *P* = 0.012) and emotional face recognition task (14.14% reduction, adjusted *P* = 0.014) significantly decreased post-training, with medium effect sizes (Cohen’s d = 0.56, 0.57). No significant changes were observed in reaction times for the Go/no-go and 0-back tasks.

### 3.6 Qualitative feedback

A thematic analysis of caregiver interviews identified five key themes, as detailed in **Table 5** with representative quotations. In response to the question, “Have you noticed improvements in any skills after the IVR training?”, the most frequently reported improvement was in social interaction and communication (N = 16), followed by emotional and behavioral regulation (N = 13). Additional improvements were observed in repetitive behaviors (N = 9), attention (N = 9), and daily life skills (N = 9).

**Table 5:** Interview themes and sample quotations (N=29).

Guiding questions and themes	N	Sample quotations
Have you noticed improvements in any skills after the IVR training?		
Social interaction and communication	16	“There’s been some improvement in social skills. For example, when he talks to us adults, he used to yell a lot, but now he yells at us less.” “I think his social skills have improved. Specifically, it feels like he smiles a bit more than before, and he jokes with me a bit more. He used to be rather cold.” “The teachers at school said he interacts more with his classmates and teachers than before. He also chats with us more at home, sharing things that upset or make him happy, which he used to keep to himself.” “His social skills have improved a little. He’s better at expressing his needs and responding to others’ requests.” “He can sense if you’re angry, upset, or sad.” “He seems a bit more sensible than two months ago and more aware of things. He can sense what adults are thinking better than before.”
Emotional and behavior regulation	13	“He seems to have better control of his emotions, and he’s been more patient with the family than before.” “We can see some progress. For example, at school, he used to have serious emotional outbursts, sometimes even getting into fights. But now, he might just throw something to vent his emotions … He himself feels that he’s controlling himself more now, whereas before, he wasn’t even aware of his emotions.” “His emotional and behavioral control seems a bit better now. Sometimes he still throws things, but before, when we told him not to, he would do the opposite. Now, he doesn’t go against us, when we tell him to do something, he does it.”
Repetitive behaviors	9	“It seems like there’s less repetition now. If we tell him not to say something again, he knows he shouldn’t say it.” “His repetitive speech has definitely decreased a lot compared to before.” “He keeps talking to me about insects. When I tell him we’ve already talked about it many times, and then he knows and stops saying it.”
Attention	8	“When doing homework, he stays focused for a bit longer than before.” “When he gets into the ‘zone’, he stays focused for longer than he used to.” “His attention has clearly improved compared to before, though I’m not sure if it’s because of the VR or a combination of factors.”
Daily life skills	8	“It seems like there was a VR scenario where the stove in the kitchen was left on, and when she’s in the kitchen, she reminds me not to forget to turn it off, saying there could be a fire and if there’s a fire, we should call 119” “There’s definitely progress. At least he’s a bit braver now. Recently, he’s been able to go out by himself, walk around, and even order food on his own.”

## 4 Discussion

The primary objective of this study is to evaluate whether IVR training can enhance adaptive skills in children and adolescents with high-functioning ASD. A key feature of this study is the development of four culturally tailored IVR training scenarios, each designed to target specific aspects of adaptive skills within a Chinese context. The training incorporates cultural elements to enhance relevance and realism. For example, the subway scenario replicates the Shanghai Metro, including actual lines and stations; the supermarket scenario includes checkout processes using widely used payment methods in China, such as Alipay, QR codes, and self-service checkout machines; the home scenario features a waste-sorting task aligned with Shanghai’s recent waste classification policies; and in the amusement park scenario, participants greet a virtual partner with the culturally familiar expression “Have you eaten?” These culturally grounded adaptations are intended to make the training experience more relatable and effective for Chinese individuals with high-functioning ASD.

### 4.1 Daily life skills

Individuals with ASD face significant challenges in daily living, for example, 59% struggling with household tasks, 30% facing difficulties using public transportation, and 38% unable to shop independently (37, 38). Less than 10% achieve independent living after graduation, with most continuing to reside with their families (39, 40). Therefore, enhancing adaptive functioning in individuals with ASD is the core objective of this study. This study employed the change in task completion time and task performance score between the first and second rounds of IVR training as the primary outcome measures, aiming to capture improvements in adaptive skills. IVR system showed a 5.5% increase in task scores (moderate effect size) and a 29.59% decrease in task completion times (large effect size) from the first to the second round, suggesting a certain degree of improvement in participants’ adaptive skills.

Previous studies have demonstrated that task completion time in IVR environments generally corresponds well to that in real-life settings (41). Thus, a reduction in task duration in the virtual environment may indicate improved efficiency in real-world performance, reflecting the transferability of skills acquired through training. Unlike prior studies that implemented only a single round of IVR training (21, 28–30), this study introduced a two-round design, allowing participants to address initial challenges and refine their skills. The substantial reduction in task time may be attributed to practice effects (42), indicating increased task fluency and processing speed.

In contrast, the smaller effect size observed in task performance scores may be related to the absence of real-world negative feedback mechanisms within the IVR system (43). The IVR environment provides a “safe” space for trial and error, which may encourage participants to explore inappropriate options without facing real-life consequences, even when they understand the correct behaviors. For instance, in the subway scenario, some participants repeatedly touched the closing subway doors without experiencing the natural consequence of being physically caught, thereby lacking the negative feedback necessary to suppress such behavior and subsequently losing points in the task. In addition, auditory warnings (e.g., alarms and voice prompts) may have unintentionally reinforced sensory-seeking behaviors commonly seen in individuals with ASD (44). Furthermore, executive function deficits—particularly in inhibitory control—frequently observed in ASD may also contribute to the limited improvement in task performance scores (45).

The improvements observed in task time and performance scores were supported to some extent by qualitative interview findings, with nine caregivers reporting perceived improvements in participants’ daily living skills. However, the secondary outcome, as measured by the ABAS-II scale, showed no significant post-training improvements across the three adaptive functioning domains. This result was expected: first, the ABAS-II comprises 232 adaptive skill items, whereas the IVR training system included only 36 tasks that targeted a relatively small subset of these skills. Naturally, skills not covered by the training program are unlikely to improve. Second, some participants may not have had the opportunity to independently apply the trained skills in real-life situations within the one-week post-training assessment window. As a result, observable behavioral changes may not have emerged within such a short timeframe.

### 4.2 Social and communication skills

Socialization and communication are crucial components of adaptive skills, and numerous studies have demonstrated effectiveness of IVR in enhancing these abilities in individuals with ASD (46–49). Children and adolescents with ASD are prone to be apprehensive and distressed about possible negative consequences of socialization (e.g., critical accusations from peers), which conversely further impede their acquisition of social skills. IVR systems, by incorporating virtual characters as social partners, provide a safe and controlled environment for them to practice, make mistakes, and learn without facing real-world consequences typically associated with social failure.

Consistent with prior studies that employ virtual characters to facilitate social interaction, the IVR system in this study features two types of virtual agents. The first type includes characters other than the virtual friend “Xiao Zhi,” such as security guards, cashiers, etc. These instrumental interactions generally consist of single conversational turns or multiple turns that lack contextual continuity, with each prompt remaining fixed regardless of the participant’s replies—such as completing specific tasks—to train daily living skills associated with those goals.

The second type is the virtual friend “Xiao Zhi,” who guides participants through emotionally-driven interactions. These interactions typically involve multiple, coherent conversational turns. Xiao Zhi responds differently depending on the participant’s selected option (usually from 2 to 4 options): appropriate responses elicit Xiao Zhi’s positive emotional and verbal feedback, while inappropriate ones result in expressions of sadness or displeasure. This design helps participants to understand emotional expressions and social feedback in various contexts, which may explain the observed improvements in relating score on the ABC scale, the reduced response time in emotional face recognition task and the reported improvements in social and communication by caregivers.

This structured, multiple-option interaction model—common in many studies involving social tasks—has several inherent limitations (50): participants’ response options are limited; the initiator of the interaction is predetermined; and regardless of the outcome of the interaction, the program proceeds to the next task step. Therefore, in the present study, a feedback phase was provided after each training phase, if participants made inappropriate choices or expressed socially inappropriate utterances during the task, the researcher would offer explanations and engage in discussions afterward to help them understand and learn more appropriate responses.

### 4.3 Executive skills and emotional regulation

Executive function (EF) refers to cognitive processes that enable individuals to consciously manage their actions and thoughts to achieve specific goals (51). EF is crucial for individuals with ASD to perform daily life skills across various settings, such as school, home, and work (52). Although some VR studies have not specifically targeted executive function—such as those focused on driving, shopping, or physical tasks—they have nonetheless reported improvements in various components of executive function (20, 53, 54). In this study, executive function was assessed through three neuropsychological tasks. Notably, reaction time in the 1-back task significantly decreased after IVR training, while no improvement was observed in the Go/no-go or 0-back tasks. This may be due to the lower cognitive load in the latter two tasks, with the 1-back task, which requires more cognitive resources, being more sensitive to IVR training. Moreover, IVR training may be particularly effective in tasks involving dynamic changes and situational responses, whereas improvements in tasks requiring simple or static reactions may be less pronounced.

Unlike the neuropsychological concept of executive function, the Executive Function Questionnaire evaluates executive skills, defined as the ability to apply executive functions across various tasks (55, 56). In this study, both the BRI and MI of BRIEF showed significant improvements following the training. The BRI reflects the ability to regulate impulses, control emotions, and shift behavior flexibly in response to internal or external challenges, whereas the MI reflects the ability to process information when performing goal-directed tasks. These results are consistent with caregivers’ interviews, with 13 noting improvements in emotional and behavioral regulation and 8 parents observing better attention control, further supporting the observed gains in executive skills.

Individuals with ASD commonly face emotional and behavioral challenges, including irritability, aggression, self-harm, anxiety, and impulsivity (57), which are often associated with deficits in emotional regulation under stress or overstimulation (58, 59). These issues may be linked to impaired inhibitory control, reduced cognitive flexibility, limited expressive language, poor problem-solving ability, difficulties in interpreting social and emotional cues, hypersensitivity to environmental changes, and biological factors (60). Among these, executive functioning plays a crucial role in regulating emotional and behavioral responses (61). The observed improvements may stem from the indirect acquisition of executive skills embedded in IVR tasks. For example, in the subway scenario, participants were required to wait patiently until the next stop; in the supermarket scenario, they needed to listen and recall the shopping list before starting the task; in the home scenario, identifying all five potential hazards was necessary to complete the danger recognition task; and in the amusement park scenario, participants had to use a map to locate destinations before proceeding. For many individuals with ASD, stress can accumulate over time, leading to task disruption or withdrawal. During IVR training, two patterns of stress response were observed: some participants gradually improved emotional and behavioral regulation through repeated practice and adaptation, while others initially refused to continue due to various obstacles. For the latter situations, researchers provided emotion regulation strategies and encouragement, ultimately enabling all participants to complete the training.

Notably, 9 caregivers reported a decrease in stereotyped speech, which may stem from improved emotion recognition—especially in response to negative emotional cues—and enhanced emotion regulation.

### 4.4 Usability

Usability could be assessed through various metrics, including completion rates, adverse effects, overall user experience, etc. (62) This study employed verbal ratings and reported feedback to dynamically capture subjective experiences while minimizing the cognitive burden of excessive testing. Among the 33 participants, 87.9% successfully completed all IVR training sessions. However, 4 participants (12.1%) withdrew early, with 3 failed to complete the tasks. Tic disorders, commonly co-occurring in individuals with ASD, can cause involuntary movements that affect device stability, leading to discomfort or reduced engagement in IVR training. For individuals exhibiting severe disruptive behaviors, pharmacological therapy, such as antipsychotics, may help stabilize symptoms before attending IVR-based training.

No severe adverse effects, such as seizures, were reported throughout the training period. However, common IVR-related discomforts were observed, including dizziness or nausea (28.6%) and headaches or fatigue (25.0%). While the reported adverse effects are consistent with those reported in previous IVR studies (63, 64), there is no established threshold for determining a safe incidence rate for such effects. In terms of device experience, a potential explanation for the discomforts like unsteady walking or falls or discomfort while wearing the device lies in the increased complexity of the IVR system used in this study. Unlike traditional VR setups that rely solely on head-mounted displays and handheld controllers, this system incorporated a treadmill with multidirectional movement. While this design enhances ecological validity by allowing free navigation, it also introduces additional physical demands, increasing the likelihood of balance issues, gait instability, and motion sickness. However, participants exhibited a general upward trend in comfort levels throughout the training, with a significant improvement from T2 to T3. This suggests a process of gradual adaptation, where repeated exposure to the IVR environment reduced sensory conflicts and increased overall tolerance.

## 5 Limitations and future directions

The evaluation of training effectiveness typically requires outcome measures that are psychometrically sound (65). However, a major limitation of this study is the lack of standardized assessment tools specifically developed for the IVR training system. Adaptive functioning encompasses a wide range of skills across developmental stages. Although this study incorporated 36 training tasks targeting commonly required adaptive skills, it remains challenging to fully capture the entire spectrum of adaptive functioning. Given that IVR simulates real-world contexts, behavioral improvements observed in the virtual environment may, to some extent, predict improvements in real-life behavior, this current study employed the change rate in task completion time and performance score during a second execution of the same task as the primary outcome measure, aiming to reflect behavioral gains. Nevertheless, this approach has notable limitations: the use of non-standardized evaluation metrics hinders comparability across studies, and the technological gap between virtual and real-world environments may limit the interpretability and generalizability of the findings. A more ideal solution would be to develop structured evaluation tools tailored to the specific content of the IVR training, with established reliability, validity, and relevance to targeted skill domains. For instance, Smith et al. designed a custom questionnaire based on the content of an interview skills training program to evaluate its effectiveness (66). Developing such tools will be a key direction for future work.

In addition to appropriate evaluation tools, the timing of follow-up assessments is also critical for accurately determining training effects. Standardized questionnaires with established psychometric properties are often completed by caregivers; however, if the follow-up is conducted too soon, caregivers may not have had sufficient time to observe noticeable improvements in the targeted skills. In this study, the post-assessment was conducted within one week after the final training session, and no long-term follow-up was performed. Therefore, incorporating multiple follow-up time points—from immediately post-training to six months post-training—would be valuable to assess both the progression and sustainability of skill changes.

Although the preliminary findings indicate that the training program is effective in certain domains, several potential confounding factors remain and warrant further investigation. Firstly, while each participant completed the same total number of tasks (two rounds of 36 tasks), the number of sessions varied depending on individual pace. Some studies suggest that shorter intervention durations may be insufficient to produce meaningful effects (67). It remains unclear whether differences in total training sessions may influence outcomes, and future studies are needed to explore this relationship. Secondly, all participants in this study were school-aged children or adolescents receiving primary or secondary education in China and were not yet required to live independently, however, considerable variation exists in age, baseline adaptive functioning, and learning ability. In addition, symptom heterogeneity and comorbid conditions may also influence outcomes. To better investigate the impact of these individual differences, larger and more diverse samples are required in future research. Thirdly, although the average training period lasted 8 weeks, observed improvements may have been contributed by natural maturation or environmental fluctuations. Therefore, future research employing randomized controlled trials (RCTs) is necessary to establish more robust evidence for the training’s effectiveness.

In terms of usability, although this study allowed participants to withdraw at any point during the training and ensured that all participation was entirely voluntary through continuous clinical monitoring, the specific factors influencing usability remain incompletely understood. Future research should incorporate standardized screening tools for sensory sensitivities, particularly assessments targeting visual and vestibular domains, to gain a deeper understanding of individual differences in tolerance and responsiveness.

## 6 Conclusion

This pre-post pilot study provided an IVR training program aimed at enhancing multiple adaptive skills in children and adolescents with high functioning ASD in various real-life settings, including subway, supermarket, home, and other common public environments. Thirty-three participants aged 8 to 18 underwent IVR training over six to ten weeks, with one-hour weekly sessions. Findings from IVR task performance, parent-reported questionnaires, interviews, and neuropsychological tests demonstrated significant improvements in daily living skills, social communication, executive functioning, emotional regulation, and mental rotation. The training was well tolerated, with a high completion rate and no severe adverse effects. Further research with standardized outcome measures, extended follow-up periods, larger and more diverse samples, and rigorous experimental designs is warranted to validate and generalize these pilot results.

## Data Availability

The original contributions presented in the study are included in the article/**Supplementary Material**. Further inquiries can be directed to the corresponding author.

## References

[1] American Psychiatric Association. Diagnostic and statistical manual of mental disorders. 5th Edition. Washington, DC: American Psychiatric Association (2013).

[2] Solmi M Song M Yon DK Lee SW Fombonne E Kim MS. Incidence, prevalence, and global burden of autism spectrum disorder from 1990 to 2019 across 204 countries. Mol Psychiatry. (2022) 27:4172–80. doi: 10.1038/s41380-022-01630-7, PMID: 35768640

[3] Christensen DL Braun KVN Baio J Bilder D Charles J Constantino JN. Prevalence and characteristics of autism spectrum disorder among children aged 8 years - autism and developmental disabilities monitoring network, 11 sites, United States, 2012. MMWR Surveill Summ. (2018) 65:1–23. doi: 10.15585/mmwr.ss6513a1, PMID: 30439868 PMC6237390

[4] Baio J Wiggins L Christensen DL Maenner MJ Daniels J Warren Z. Prevalence of autism spectrum disorder among children aged 8 years - autism and developmental disabilities monitoring network, 11 sites, United States, 2014. MMWR Surveill Summ. (2018) 67:1–23. doi: 10.15585/mmwr.ss6706a1, PMID: 29701730 PMC5919599

[5] Maenner MJ Shaw KA Bakian AV Bilder DA Durkin MS Esler A. Prevalence and characteristics of autism spectrum disorder among children aged 8 years - autism and developmental disabilities monitoring network, 11 sites, United States, 2018. MMWR Surveill Summ. (2021) 70:1–16. doi: 10.15585/mmwr.ss7011a1, PMID: 34855725 PMC8639024

[6] Maenner MJ Warren Z Williams AR Amoakohene E Bakian AV Bilder DA. Prevalence and characteristics of autism spectrum disorder among children aged 8 years - autism and developmental disabilities monitoring network, 11 sites, United States, 2020. MMWR Surveill Summ. (2023) 72:1–14. doi: 10.15585/mmwr.ss7202a1, PMID: 36952288 PMC10042614

[7] Zhou H Xu X Yan W Zou X Wu L Luo X. Prevalence of autism spectrum disorder in China: A nationwide multi-center population-based study among children aged 6 to 12 years. Neurosci Bull. (2020) 36:961–71. doi: 10.1007/s12264-020-00530-6, PMID: 32607739 PMC7475160

[8] Jiang X Chen X Su J Liu N. Prevalence of autism spectrum disorder in mainland China over the past 6 years: A systematic review and meta-analysis. BMC Psychiatry. (2024) 24:404. doi: 10.1186/s12888-024-05729-9, PMID: 38811881 PMC11137880

[9] Tassé MJ Schalock RL Balboni G Bersani H Jr. Borthwick-Duffy SA Spreat S. The construct of adaptive behavior: its conceptualization, measurement, and use in the field of intellectual disability. Am J Intellect Dev Disabil. (2012) 117:291–303. doi: 10.1352/1944-7558-117.4.291, PMID: 22809075

[10] Alvares GA Bebbington K Cleary D Evans K Glasson EJ Maybery MT. The misnomer of ‘High functioning autism’: intelligence is an imprecise predictor of functional abilities at diagnosis. Autism. (2020) 24:221–32. doi: 10.1177/1362361319852831, PMID: 31215791

[11] Farley MA McMahon WM Fombonne E Jenson WR Miller J Gardner M. Twenty-year outcome for individuals with autism and average or near-average cognitive abilities. Autism Res. (2009) 2:109–18. doi: 10.1002/aur.69, PMID: 19455645

[12] McCauley JB Pickles A Huerta M Lord C. Defining positive outcomes in more and less cognitively able autistic adults. Autism Res. (2020) 13:1548–60. doi: 10.1002/aur.2359, PMID: 32851813

[13] Clarke EB McCauley JB Lord C. Post-high school daily living skills in autism spectrum disorder. J Am Acad Child Adolesc Psychiatry. (2021) 60:978–85. doi: 10.1016/j.jaac.2020.11.008, PMID: 33220430 PMC8128936

[14] Syriopoulou-Delli CK Sarri K. Video-based instruction in enhancing functional living skills of adolescents and young adults with autism spectrum disorder and their transition to independent living: A review. Int J Dev Disabil. (2022) 68:788–99. doi: 10.1080/20473869.2021.1900504, PMID: 36568614 PMC9788681

[15] Järbrink K McCrone P Fombonne E Zandén H Knapp M. Cost-impact of young adults with high-functioning autistic spectrum disorder. Res Dev Disabil. (2007) 28:94–104. doi: 10.1016/j.ridd.2005.11.002, PMID: 16551499

[16] Leigh JP Du J. Brief report: forecasting the economic burden of autism in 2015 and 2025 in the United States. J Autism Dev Disord. (2015) 45:4135–9. doi: 10.1007/s10803-015-2521-7, PMID: 26183723

[17] Strickland DC Coles CD Southern LB. JobTIPS: a transition to employment program for individuals with autism spectrum disorders. J Autism Dev Disord. (2013) 43:2472–83. doi: 10.1007/s10803-013-1800-4, PMID: 23494559 PMC3706489

[18] Genova HM Lancaster K Morecraft J Haas M Edwards A Dibenedetto M. A pilot RCT of virtual reality job interview training in transition-age youth on the autism spectrum. Res Autism Spectr Disord. (2021) 89:101878. doi: 10.1016/j.rasd.2021.101878 PMC12435450 40958891

[19] Smith MJ Sherwood K Ross B Smith JD DaWalt L Bishop L. Virtual interview training for autistic transition age youth: A randomized controlled feasibility and effectiveness trial. Autism. (2021) 25:1536–52. doi: 10.3102/1693269 PMC8324503 33567883

[20] Lamash L Klinger E Josman N eds. (2017). Using a virtual supermarket to promote independent functioning among adolescents with autism spectrum disorder, in: 2017 International Conference on Virtual Rehabilitation (ICVR). Montreal.

[21] Simões M Bernardes M Barros F Castelo-Branco M. Virtual travel training for autism spectrum disorder: proof-of-concept interventional study. JMIR Serious Games. (2018) 6:e5. doi: 10.2196/games.8428, PMID: 29559425 PMC5883078

[22] Freeman D Reeve S Robinson A Ehlers A Clark D Spanlang B. Virtual reality in the assessment, understanding, and treatment of mental health disorders. Psychol Med. (2017) 47:2393–400. doi: 10.1017/S003329171700040X, PMID: 28325167 PMC5964457

[23] Shibata T. Head mounted display. Displays. (2002) 23:57–64. doi: 10.1016/S0141-9382(02)00010-0

[24] Burdea G Coiffet P. Virtual reality technology. Hoboken: Wiley Interscience (2003).

[25] Slater M Wilbur SA. Framework for Immersive Virtual Environments (FIVE): speculations on the role of presence in virtual environments. presence teleoperators & virtual environments. (1997) 6(6):603–16. doi: 10.1162/pres.1997.6.6.603

[26] Church BA Rice CL Dovgopoly A Lopata CJ Thomeer ML Nelson A. Learning, plasticity, and atypical generalization in children with autism. Psychon Bull Rev. (2015) 22:1342–8. doi: 10.3758/s13423-014-0797-9, PMID: 25561418

[27] Berggren S Fletcher-Watson S Milenkovic N Marschik PB Bölte S Jonsson U. Emotion recognition training in autism spectrum disorder: A systematic review of challenges related to generalizability. Dev Neurorehabil. (2018) 21:141–54. doi: 10.1080/17518423.2017.1305004, PMID: 28394669

[28] Dixon DR Miyake CJ Nohelty K Novack MN Granpeesheh D. Evaluation of an immersive virtual reality safety training used to teach pedestrian skills to children with autism spectrum disorder. Behav Anal Practice. (2020) 13:631–40. doi: 10.1007/S40617-019-00401-1, PMID: 32953391 PMC7471232

[29] Miller IT Wiederhold BK Miller CS Wiederhold MD. Virtual reality air travel training with children on the autism spectrum: A preliminary report. Cyberpsychology Behavior Soc Networking. (2019) 23:10–5. doi: 10.1089/cyber.2019.0093, PMID: 31355673

[30] Miller IT Miller CS Wiederhold MD Wiederhold BK. Virtual reality air travel training using apple iPhone X and google cardboard: A feasibility report with autistic adolescents and adults. Autism Adulthood. (2020) 2:325–33. doi: 10.1089/aut.2019.0076, PMID: 36600956 PMC8992860

[31] Lord C Rutter M Le Couteur A. Autism diagnostic interview-revised: A revised version of a diagnostic interview for caregivers of individuals with possible pervasive developmental disorders. J Autism Dev Disord. (1994) 24:659–85. doi: 10.1007/BF02172145, PMID: 7814313

[32] Wang YZ J Cui JF Fan HZ Chen N Yao J Duan JH. Reliability and construct validity of the Chinese version of the Wechsler Adult Intelligence Scale–Fourth Edition. Chin Ment Health J. (2013) 27:692–7. doi: 10.3969/j.issn.1000-6729.2013.09.011

[33] Chen H Keith TZ Weiss L Zhu J Li Y. Testing for multigroup invariance of second-order WISC-IV structure across China, hong kong, macau, and Taiwan. Pers Individ Differences. (2010) 49:677–82. doi: 10.1016/j.paid.2010.06.004

[34] Oakland T Harrison PL. Adaptive behavior assessment system-II. Pittsburgh: Academic Press (2008).

[35] Jian-Ping LU Zhi-Wei Y Ming-Yao S Lin-Yan SU. Reliability, validity analysis of the childhood autism rating scale. China J Modern Med. (2004) 14:119–23. doi: 1005-8982(2024)13-0119-03

[36] Qian Y Wang Y-F. Reliability and validity of behavior rating scale of executive function parent form for school age children in China. Beijing da xue xue bao Yi xue ban = J Peking Univ Health Sci. (2007) 39:277–83. doi: 10.19723/j.issn.1671-167x.2007.03.015, PMID: 17572784

[37] De Schipper E Lundequist A Coghill D De Vries PJ Granlund M Holtmann M. Ability and disability in autism spectrum disorder: A systematic literature review employing the international classification of functioning, disability and health-children and youth version. Autism Res. (2015) 8:782–94. doi: 10.1002/aur.1485, PMID: 25820780 PMC6680328

[38] Mahdi S Albertowski K Almodayfer O Arsenopoulou V Carucci S Dias JC. An international clinical study of ability and disability in autism spectrum disorder using the WHO-ICF framework. J Autism Dev Disord. (2018) 48:2148–63. doi: 10.1007/s10803-018-3482-4, PMID: 29423605 PMC5948258

[39] Wehman P Schall C Carr S Targett P West M Cifu G. Transition from school to adulthood for youth with autism spectrum disorder: what we know and what we need to know. J Disability Policy Stud. (2014) 25:30–40. doi: 10.1177/1044207313518071

[40] Howlin P Goode S Hutton J Rutter M. Adult outcome for children with autism. J Child Psychol Psychiatry. (2004) 45:212–29. doi: 10.1111/j.1469-7610.2004.00215.x, PMID: 14982237

[41] Schmidt M Glaser N Schmidt C Kaplan R Palmer H Cobb S. Programming for generalization: confronting known challenges in the design of virtual reality interventions for autistic users. Comput Education: X Reality. (2023) 2:100013. doi: 10.1080/13854046.2012.680913, PMID: 22540222

[42] Calamia M Markon K Tranel D. Scoring higher the second time around: meta-analyses of practice effects in neuropsychological assessment. Clin Neuropsychologist. (2012) 26:543–70. doi: 10.1080/13854046.2012.680913, PMID: 22540222

[43] Price S Jewitt C Chubinidze D Barker N Yiannoutsou N. Taking an extended embodied perspective of touch: connection-disconnection in iVR. Front. Virtual Real. (2021) 2:642782. doi: 10.3389/frvir.2021.642782

[44] Zachor DA Ben-Itzchak E. The relationship between clinical presentation and unusual sensory interests in autism spectrum disorders: A preliminary investigation. J Autism Dev Disord. (2014) 44:229–35. doi: 10.1007/s10803-013-1867-y, PMID: 23797714

[45] Demetriou EA Lampit A Quintana DS Naismith SL Song YJC Pye JE. Autism spectrum disorders: A meta-analysis of executive function. Mol Psychiatry. (2017) 23:1198–204. doi: 10.1038/mp.2017.75, PMID: 28439105 PMC5984099

[46] Calderone A Militi A Latella D De Luca R Corallo F De Pasquale P. Harnessing virtual reality: improving social skills in adults with autism spectrum disorder. J Clin Med. (2024) 13(21):6435. doi: 10.3390/jcm13216435, PMID: 39518573 PMC11546170

[47] Astafeva D Syunyakov T Shapievskii D Malashonkova E Vlasov A Shport S. Virtual reality/augmented reality (VR/AR) approach to develop social and communication skills in children and adolescents with autism spectrum disorders without intellectual impairment. Psychiatr Danub. (2024) 36:361–70., PMID: 39378497

[48] Dechsling A Orm S Kalandadze T Sütterlin S Øien RA Shic F. Virtual and augmented reality in social skills interventions for individuals with autism spectrum disorder: A scoping review. J Autism Dev Disord. (2022) 52:4692–707. doi: 10.1007/s10803-021-05338-5, PMID: 34783991 PMC9556391

[49] Capobianco M Puzzo C Di Matteo C Costa A Adriani W. Current virtual reality-based rehabilitation interventions in neuro-developmental disorders at developmental ages. Front Behav Neurosci. (2024) 18:1441615. doi: 10.3389/fnbeh.2024.1441615, PMID: 39882439 PMC11775633

[50] Zhang M Ding H Naumceska M Zhang Y. Virtual reality technology as an educational and intervention tool for children with autism spectrum disorder: current perspectives and future directions. Behav Sci (Basel). (2022) 12:138. doi: 10.3390/bs12050138, PMID: 35621435 PMC9137951

[51] Cristofori I Cohen-Zimerman S Grafman J. Handbook of clinical neurology-executive functions. Amsterdam: Elsevier (2019). 10.1016/B978-0-12-804281-6.00011-2 31590731

[52] Gardiner E Iarocci G. Everyday Executive Function Predicts Adaptive and Internalizing Behavior among Children with and without Autism Spectrum Disorder. Autism Res. (2018) 11:284–95. doi: 10.1002/aur.1877, PMID: 28960841

[53] Sepehri Bonab H Ebrahimi Sani S Behzadnia B. The impact of virtual reality intervention on emotion regulation and executive functions in autistic children. Games Health J. (2024) 14(2):146–58. doi: 10.1089/g4h.2023.0240, PMID: 39109573

[54] Cox DJ Brown T Ross V Moncrief M Schmitt R Gaffney G. Can youth with autism spectrum disorder use virtual reality driving simulation training to evaluate and improve driving performance? An exploratory study. J Autism Dev Disord. (2017) 47:2544–55. doi: 10.1007/s10803-017-3164-7, PMID: 28540452

[55] Diamond A Lee K. Interventions shown to aid executive function development in children 4 to 12 years old. Science. (2011) 333:959–64. doi: 10.1126/science.1204529, PMID: 21852486 PMC3159917

[56] Qian Y Chen M Shuai L Cao QJ Yang L Wang YF. Effect of an ecological executive skill training program for school-aged children with attention deficit hyperactivity disorder: A randomized controlled clinical trial. Chin Med J (Engl). (2017) 130:1513–20. doi: 10.4103/0366-6999.208236, PMID: 28639564 PMC5494912

[57] Lecavalier L. Behavioral and emotional problems in young people with pervasive developmental disorders: relative prevalence, effects of subject characteristics, and empirical classification. J Autism Dev Disord. (2006) 36:1101–14. doi: 10.1007/s10803-006-0147-5, PMID: 16897387

[58] Laurent AC Rubin E. Challenges in emotional regulation in asperger syndrome and high-functioning autism. Topics Lang Disord. (2004) 24:271–85. doi: 10.1097/00011363-200410000-00006

[59] Konstantareas MM Stewart K. Affect regulation and temperament in children with autism spectrum disorder. J Autism Dev Disord. (2006) 36:143–54. doi: 10.1007/s10803-005-0051-4, PMID: 16456727

[60] Mazefsky CA White SW. Emotion regulation: concepts & Practice in autism spectrum disorder. Child Adolesc Psychiatr Clin N Am. (2014) 23:15–24. doi: 10.1016/j.chc.2013.07.002, PMID: 24231164 PMC3830422

[61] Costescu C Roșan A David C Cozma L Calota A. The relation between cognitive and emotional processes in children and adolescents with neurodevelopmental disorders-A meta-analysis. Eur J Investig Health Psychol Educ. (2023) 13:2811–26. doi: 10.3390/ejihpe13120194, PMID: 38131893 PMC10742924

[62] Yi YJ Heidari Matin N Brannan D Johnson M Nguyen A. Design considerations for virtual reality intervention for people with intellectual and developmental disabilities: A systematic review. Herd. (2024) 17:212–41. doi: 10.1177/19375867241271434, PMID: 39155566 PMC11608522

[63] McCleery JP Zitter A Solórzano R Turnacioglu S Miller JS Ravindran V. Safety and feasibility of an immersive virtual reality intervention program for teaching police interaction skills to adolescents and adults with autism. Autism Res. (2020) 13:1418–24. doi: 10.1002/aur.2352, PMID: 32762029

[64] Ravindran V Osgood M Sazawal V Solorzano R Turnacioglu S. Virtual reality support for joint attention using the floreo joint attention module: usability and feasibility pilot study. JMIR Pediatr Parent. (2019) 2:e14429. doi: 10.2196/14429, PMID: 31573921 PMC6792024

[65] Fok CC Henry D. Increasing the sensitivity of measures to change. Prev Sci. (2015) 16:978–86. doi: 10.1007/s11121-015-0545-z, PMID: 25703381 PMC4547914

[66] Smith MJ Ginger EJ Wright K Wright MA Taylor JL Humm LB. Virtual reality job interview training in adults with autism spectrum disorder. J Autism Dev Disord. (2014) 44:2450–63. doi: 10.1007/s10803-014-2113-y, PMID: 24803366 PMC4167908

[67] Budimirovic DB Berry-Kravis E Erickson CA Hall SS Hessl D Reiss AL. Updated report on tools to measure outcomes of clinical trials in fragile X syndrome. J Neurodev Disord. (2017) 9:14. doi: 10.1186/s11689-017-9193-x, PMID: 28616097 PMC5467057

