# Supplementary material for: Effects of immersive virtual reality training on the adaptive skills of children and adolescents with high functioning autism spectrum disorder: a mixed-methods pre-post study
Source: Front Psychiatry. 2025 Aug 18;16:1570437. doi: 10.3389/fpsyt.2025.1570437 (PMC12399565; doi:10.3389/fpsyt.2025.1570437)
Supplement: Supplementary file 1 [file SupplementaryFile1.docx]

Supplementary Material

# Demonstration of Immersive Virtual Reality Tasks

The video includes screen recordings of each scenario on an external display, as well as the performance of the task by a researcher. It should be noted that the field of view on the external display is smaller than that of the head-mounted display.

# Neuropsychological Tests

Go/no-go task (see Figure S1). This task was designed to assess inhibition ability. In each trial, an image of either a lion or a giraffe was randomly displayed on the screen. Participants were instructed to press the left mouse button as quickly as possible when a lion appeared (go trials) and to withhold their response when a giraffe appeared (no-go trials). The task comprised three blocks of 24 trials each, with 12 presentations of lions and 12 of giraffes per block. Each trial lasted 1000 ms, including a 200 ms image presentation followed by an 800 ms blank screen.


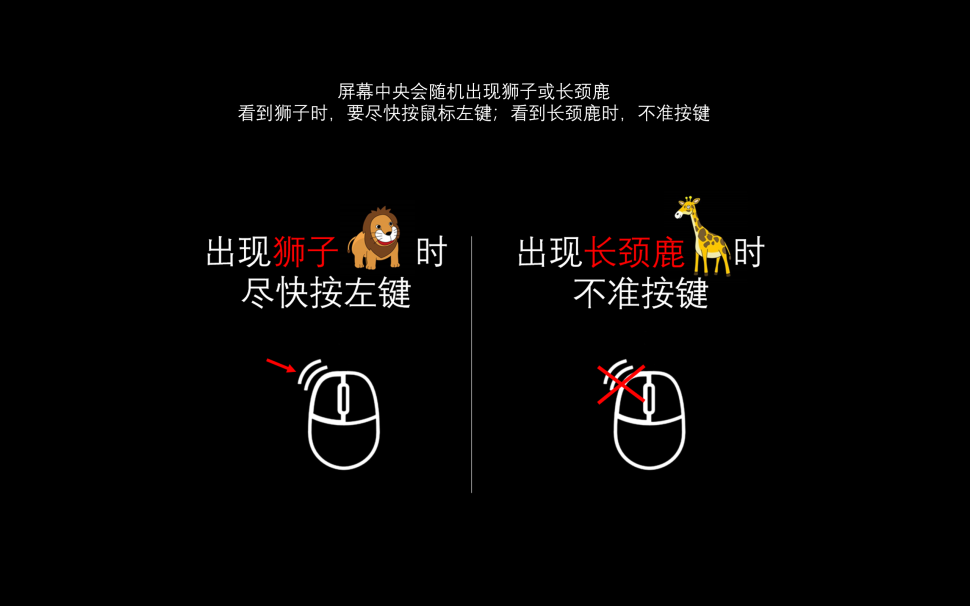


Figure S1 Instructional interface of go/no-go task

0-back task (see Figure S2). This task was designed to assess working memory, specifically the ability to retain and process information over a short period. In each trial, a random number was displayed on the screen. Participants were instructed to press the left mouse button as quickly as possible when the target number appeared and the right mouse button for non-target numbers. The task consisted of three blocks of 15 trials each, with two target number trials and 13 non-target number trials per block. Each trial lasted 2000 ms, including a 300 ms number presentation followed by a 1700 ms blank screen.


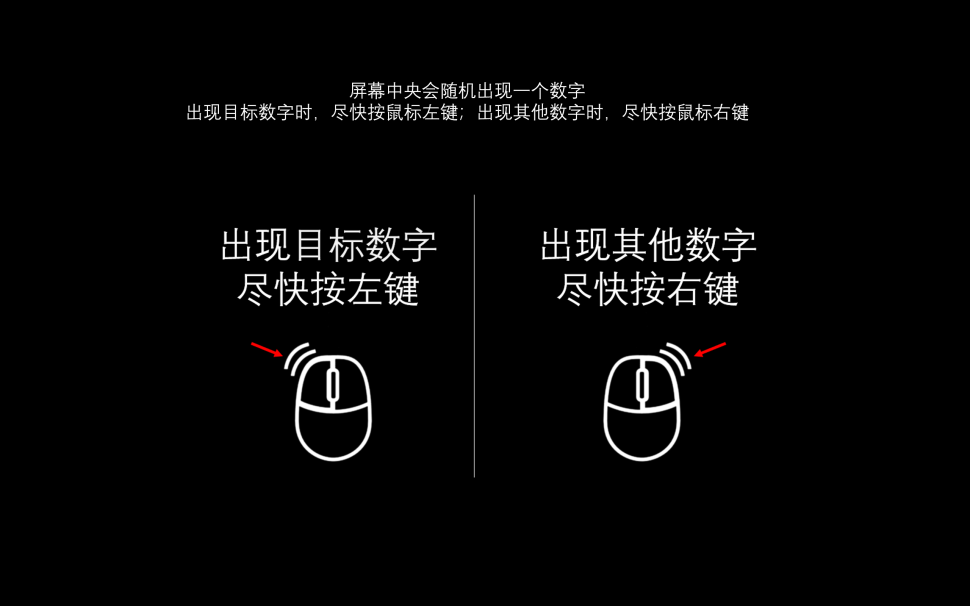


Figure S2 Instructional interface of 0-back task

1-back task (see Figure S3). This task was designed to assess working memory under increased cognitive load compared to the 0-Back task. In each trial, a random number was displayed on the screen. Participants were required to quickly determine whether the current number matched the previous one, pressing the left mouse button if the numbers were the same and the right mouse button if they were different. The task consisted of three blocks of 15 trials each, including two repeated number trials and 13 non-repeated number trials per block. Each trial lasted 2000 ms, with a 300 ms number presentation followed by a 1700 ms blank screen.


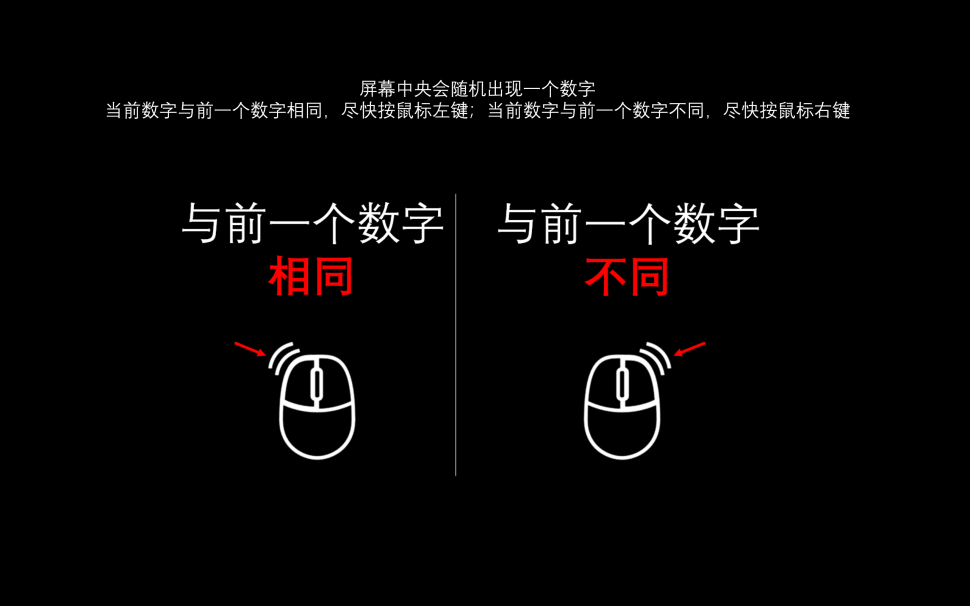


Figure S3 Instructional interface of 1-back task

Emotional face recognition task (see Figure S4). This task was designed to assess social cognition, specifically the ability to recognize and interpret others' emotions. At the start of each trial, an image of a face displaying a specific emotion was presented at the top of the screen. Participants were required to identify which of the two faces displayed at the bottom of the screen matched the emotion of the top image, pressing the left mouse button for the left face and the right mouse button for the right face. The task consisted of three blocks of 12 trials each, with a maximum trial duration of 4500 ms. The face image was displayed for 4000 ms, followed by a 500 ms blank screen. If a choice was made before the time limit, the trial immediately proceeded to the blank screen.


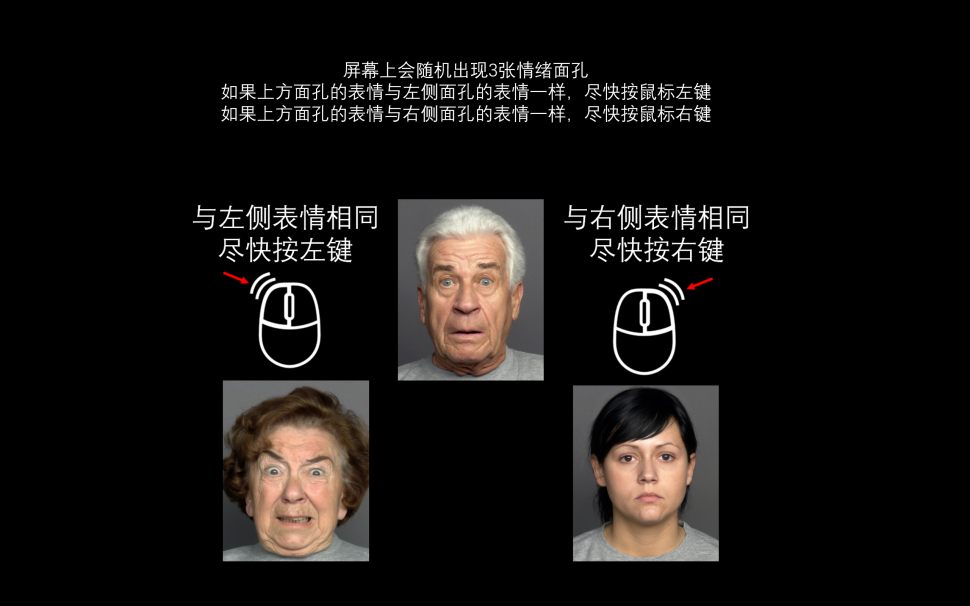


Figure S4 Instructional interface of emotional face recognition task
